# Supplementary material for: The RMaP challenge of predicting RNA modifications by nanopore sequencing
Source: Commun Chem. 2025 Apr 12;8:115. doi: 10.1038/s42004-025-01507-0 (PMC11993749; doi:10.1038/s42004-025-01507-0)
Supplement: Supplementary file 2 — Supplementary Material [file 42004_2025_1507_MOESM2_ESM.pdf]

Predicting RNA modifications by nanopore sequencing: The RMaP challenge

Supplementary note

Reference Sequence for Challenge 1

GAAACAAATCATGGCGGTACTATTCTTAGCAGGACGAGTCGTTGCTGAACAGGT
CTAAGCATTACTGATCGATGCTTGACGGTTCAAGGCGTAACATTTCAGTGCAATA
CTTGTCTGGGCTAGACGTATCGGAGCGAAACGAATCGTGGCTGTACAATTCATA
GCGGGACTAGTCTTTGCAGAACGGGTCAAAGCGTTACAGATCTATGCATGACTG
TTCGAGGCTTAACGTTTCGGTGCGATACATGTCAGGGCAAGACTTATCTGAGCTA
AACTAATCTTGGCAGTACGATTCGTAGCTGGACAAGTCATTGCGGAACTGGTCG
AAGCTTTACGGATCAATGCGTGACAGTTCTAGGCATAACTTTTCTGTGCTATACGT
GTCGGGGCGAGACATATCAGAGCAAAACAAGGCGGTTCTTGACGATGCTGATCA
TTACTAAGCAGGTCGTAACAGTGCTTGTCTAGACGGAGCATTTCAATACTGGGCG
TATCGAAACATAGCGAATCGGGACGTGGCTAGTCTGTACTTTGCAATTCAGAACG
AGGCTGTTCATGACTATGCAGATCGTTACAAAGCGGGTCTTAACGGTGCATGTCA
AGACTGAGCGTTTCGATACAGGGCTTATCTAAACGTAGCTAATCTGGACTTGGCA
AGTCAGTACATTGCGATTCGGAACTAGGCAGTTCGTGACAATGCGGATCTTTACG
AAGCTGGTCATAACTGTGCGTGTCGAGACAGAGCTTTTCTATACGGGGCATATCA
AAACTTAGCAAATCAGGACATGGCGAGTCGGTACGTTGCTATTCTGAACAATTCT
TTGCTGTACTAGTCGTGGCGGGACGAATCATAGCAGAACAGATCGAGGCGTTAC
TGTTCAAAGCATGACGGGTCTATGCTTAACATGTCTGAGCGATACTTATCGGTGC
AAGACGTTTCAGGGCTAAACGATTCATTGCAGTACAAGTCTTGGCTGGACTAATC
GTAGCGGAACGGATCTAGGCTTTACAGTTCGAAGCGTGACTGGTCAATGCATAA
CGTGTCAGAGCTATACATATCTGTGCGAGACTTTTCGGGGCAAAACTATTCGTTG
CGGTACGAGTCATGGCAGGACAAATCTTAGCTGAACTGATCAAGGCATTACGGT
TCTAAGCTTGACAGGTCGATGCGTAACTTGTCGGAGCAATACGTATCAGTGCTAG
ACATTTCTGGGCGAAA

The DNA sequence for Challenge 1 containing all 5-mers with a single C in the middle of the 5-mer.

Reference Sequence for Challenge 2

GCCCACCCTACTGGAGGTCATCTTATTCGACGGCAGCGTAGTTGATGCCACGGT
ATCCGACTTCATGCTAGCTGATTGCAGGTTACCGGAGTCCACTTTACGTGACCTC
ATTGTATGGGATCGCAGTCTAGGCGAGCCCAGCCTAGTGGATGTCACCTTACTC
GAGGGCATCGTATTTGACGCCAGGGTACCCGAGTTCACGCTATCTGACTGCATG
TTAGCGGATTCCAGTTTAGGTGAGCTCACTGTACGGGACCGCATTCTATGCGATC
CCATCCTATTGGACGTCAGCTTAGTCGATGGCACCGTACTTGAGGCCATGGTAG
CCGATTTCAGGCTACCTGAGTGCACGTTATCGGACTCCATTTTATGTGATCTCAG
TGTAGGGGAGCGCACTCTACGCGACCCCACCGGAGGTTATTGCAGCTGATGCT
ACTTCATCCGACGGTAGTCCACGTGATTGTATCGCAGGCGACTTTACCTCATGG

*GAGTCTAGCCCACTCGAGCCTAGGGCAGTGGATCGTATGTCATTTGACCTTACG
CCAGCGGATGTTACTGCATCTGACGCTAGTTCACCCGAGGGTATTCCAGGTGAC
TGTACCGCATGCGAGTTTAGCTCACGGGATTCTATCCCAGTCGATCCTATGGCAT
TGGACCGTACGTCACTTGAGCTTAGGCCATCGGACGTTAGTGCACCTGAGGCTA
TTTCAGCCGATGGTACTCCATGTGAGTGTAGCGCACGCGATTTTATCTCAGGGG
ACTCTACCCCATTCGACCCTACGGCACTGGAGCGTAGGTCAGTTGATCTTATGC
CACCTTATTTGATGTCATCGTAGTGGAGGGCAGCCTACTCGACGCCACGCTAGC
GGAGTTCATGTTACCCGACTGCAGGGTATCTGATTCCACTGTATGCGAGCTCATT
CTAGGTGACCGCAGTTTACGGGATCCCAGCTTACTTGACGTCACCGTATTGGAT
GGCATCCTAGTCGAGGCCAGGCTATCGGATTTCACGTTAGCCGAGTGCATGGTA
CCTGACTCCAGTGTACGCGATCTCACTCTATGTGAGCGCATTTTAGGGGACCCC
ATCTTAGTTGAGGTCAGCGTACTGGACGGCACCCTATTCGATGCCATGCTACCG
GACTTCAGGTTATCCGATTGCACGGTAGCTGAGTCCATTGTAGGCGACCTCAGT
CTACGTGATCGCACTTTATGGGAGCCC*

The DNA sequence for Challenge 2 containing all 5-mers with a single A in the middle of the 5-mer.

Reference Sequence for Challenge 3

*CAAATAAAGTAGCCTCCGATGAGGTGGACTACCATCACGTCGGCTGCAATACCG
TGAACTAGGATGCAGTCAGCTGGCATCCGGTAACCTCGAATAGGGTACGCTAAG
ATGGCGTGCCCTGACATCGAGTCCACTCAAATCAAGTCGCCTGCGATAAGGTAG
ACTCCCATGACGTGGGCTACAATCCCGTAAACTCGGATACAGTGAGCTAGCATG
CGGTCACCTGGAATCGGGTCCGCTCAGATAGCGTACCCTAACATGGAGTGCACT
GAAATGAAGTGGCCTACGATCAGGTCGACTGCCATAACGTAGGCTCCAATGCCG
TCAACTGGGATCCAGTAAGCTCGCATACGGTGACCTAGAATGGGGTGCGCTGA
GATCGCGTCCCCTCACATAGAGTACACTAAAATAACCTCCGGTGGCATCAGCTG
CAGTAGGATGAACTACCGTCGAATACGCTGGCGTGACATCCACTAGGGTAAGAT
GCCCTCGAGTCAAATAGACTCAAGTCCCATCGCCTGACGTGCGATGGGCTAAG
GTACAATCACCTGCGGTAGCATGAGCTACAGTCGGATAAACTCCCGTGGAATCC
GCTAGCGTAACATGCACTCGGGTCAGATACCCTGGAGTGAAATCGACTGAAGTG
CCATGGCCTAACGTACGATAGGCTCAGGTCCAATGACCTACGGTCGCATAAGCT
CCAGTGGGATCAACTGCCGTAGAATGCGCTCGCGTCACATACACTGGGGTGAG
ATCCCCTAGAGTAAAATGGACTAAAGTACCATAGCCTCACGTCCGATCGGCTGAG
GTGCAATAAGGTGGGCTGCGATGACGTCGCCTCCCATCAAGTAGACTACAATAC
AGTCACCTCGGATGCGGTAAACTAGCATCCCGTGAGCTGGAATAGCGTGCACTC
AGATGGAGTCCGCTAACATCGGGTACCCTGAAATCAGGTAGGCTACGATAACGT
GGCCTGCCATGAAGTCGACTCCAATCCAGTGACCTGGGATACGGTCAACTCGC
ATGCCGTAAGCTAGAATCGCGTACACTGAGATAGAGTGCGCTCACATGGGGTCC
CCTAAAATGAGGTCGGCTCCGATCACGTAGCCTACCATAAAGTGGACTGCAATG
CAGTAACCTAGGATCCGGTGAACTGGCATACCGTCAGCTCGAATGGCGTCCACT
AAGATCGAGTACGCTGACATAGGGTGCCCTCAAA*

The DNA sequence for Challenge 3 containing all 5-mers with a single U in the middle of the 5-mer.
